# Groove rhythm stimulates prefrontal cortex function in groove enjoyers

**DOI:** 10.1038/s41598-022-11324-3

**Published:** 2022-05-05

**Authors:** Takemune Fukuie, Kazuya Suwabe, Satoshi Kawase, Takeshi Shimizu, Genta Ochi, Ryuta Kuwamizu, Yosuke Sakairi, Hideaki Soya

**Affiliations:** 1 Laboratory of Exercise Biochemistry and Neuroendocrinology, Faculty of Health and Sport Sciences, University of Tsukuba, Tsukuba, Ibaraki 305-8574 Japan; 2 Sports Neuroscience Division, Department of Mind, Advanced Research Initiative for Human High Performance (ARIHHP), Faculty of Health and Sport Sciences, University of Tsukuba, Tsukuba, Ibaraki 305-8574 Japan; 3 Faculty of Health and Sport Sciences, Ryutsu Keizai University, Ryūgasaki, Ibaraki 301-8555 Japan; 4 Faculty of Psychology, Kobe Gakuin University, Kobe, Hyogo 651-2180 Japan; 5 School of Information and Communication, Meiji University, Chiyoda-ku, Tokyo 101-8301 Japan; 6 Department of Health and Sports, Niigata University of Health and Welfare, Niigata, Niigata 950-3198 Japan

**Keywords:** Attention, Auditory system, Cognitive neuroscience, Emotion, Neuroscience, Depression

## Abstract

Hearing a groove rhythm (GR), which creates the sensation of wanting to move to the music, can also create feelings of pleasure and arousal in people, and it may enhance cognitive performance, as does exercise, by stimulating the prefrontal cortex. Here, we examined the hypothesis that GR enhances executive function (EF) by acting on the left dorsolateral prefrontal cortex (l-DLPFC) while also considering individual differences in psychological responses. Fifty-one participants underwent two conditions: 3 min of listening to GR or a white-noise metronome. Before and after listening, participants performed the Stroop task and were monitored for l-DLPFC activity with functional near-infrared spectroscopy. Our results show that GR enhanced EF and l-DLPFC activity in participants who felt a greater groove sensation and a more feeling clear-headed after listening to GR. Further, these psychological responses predict the impact of GR on l-DLPFC activity and EF, suggesting that GR enhances EF via l-DLPFC activity when the psychological response to GR is enhanced.

## Introduction

A growing amount of evidence shows that physical exercise has beneficial effects on cognitive functions^1^, especially on human executive function (EF) mainly in the prefrontal cortex (PFC)^2^. Our recent study using acute exercise with music showed that the key factor of exercise’s effect on prefrontal executive function is a positive affective response^3^ expressed the two-dimensional axis of pleasure and arousal.

Another potential stimulus which can improve EF through a positive affective response is groove rhythm (GR). GR is a musical rhythm that induces the sensation of “Wanting to move to the music” (groove sensation) accompanied by positive affective responses while listening to music^4,5^. GR can be defined by the subjective score of “Wanting to move to the music” and “Good *nori*”^4,6^. The groove sensation can be modulated by syncopation. Syncopation is a method of shifting rhythmic emphasis by manipulating the complexity of a rhythm. Rhythm with low to medium syncopation induces a higher groove sensation than does rhythm with high syncopation as evidenced by averages of mass groups in previous studies^7–9^. Low-frequency components, such as the bass drum, induce entrainment of body movement and musical beat^10^. In several previous studies, drum breaks consisting of hi-hat, snare-drum, and bass-drum sounds were used because they made it easy to control rhythmic factors, syncopation, bass sound, and tempo^7–12^.

Moving the body to music is a universal phenomenon (e.g., clapping, nodding, swaying) and one of the main powers of music^13^. Music with groove-inducing characteristics increasing in recent music popularity charts may be indicative of this^14^. That groove music improves gait performance in Parkinson’s disease (PD) by reducing the cognitive demands of synchronizing to the beat and promoting vigorous movement^15–17^ shows that groove music affects the interaction between body movement and brain function. More interestingly, listening to groove music induces entrainment of body movement and musical rhythm together with positive affective responses^18^ and activates neural networks associated with motor and reward systems^11,19,20^. Since the dopaminergic reward system projects not only to emotion-related brain areas but also to cognition-related areas such as the PFC^21–23^, GR could increase PFC activity and lead to improved EF. However, no research has explored the effect of groove music on EF and prefrontal activity to date. The reason for this could be that the effect of GR on EF may have large individual differences because both groove sensation and concurrent positive affective responses to groove music would have many individual differences^8,24^ and both responses are associated with reward system activity^11^. Therefore, we should examine the single effects of GR on EF and its relationship with PFC activity. To examine this, consideration of the psychological responses to listening to GR as influential factors that explain individual differences and creation of an experimental model which can evaluate the effect of GR on EF and how it is related to PFC activity are necessary.

To that end, in the current study we introduce the combination of an acute experimental model that was used for the detection of exercise’s effect on cognition and prefrontal activity and a grouping analysis to explore the psychological response to GR. In our previous research, we used functional near-infrared spectroscopy (fNIRS) with the color-word-matching Stroop task (CWST)^25,26^, which evaluates inhibitory EF, in order to clarify the effects of an acute bout (10 min) of exercise^27–31^. fNIRS is a non-invasive neuroimaging method which can monitor hemodynamic response to neural activation (neurovascular coupling)^32^ by using near-infrared light passing through tissue. Since fNIRS allows for the least restrictive measuring environment among neuroimaging modalities, it can measure regional cortical activation boosted by listening to music while minimizing possible negative environmental influences on psychological response and cognition. The CWST has been adopted in numerous neuroimaging studies including fNIRS studies^33–35^, and the brain regions related with the task are well known. The DLPFC is a key region for inhibitory control of EF^36^ and responsible for CWST performance^37,38^. In addition, the left hemisphere plays a key role in the processing of verbal information^39–42^. Therefore, we focused on the left dorsolateral prefrontal cortex (l-DLPFC) as the region of interest (ROI). A previous study indicated that l-DLPFC activity correlated with a positive affective response and that EF changed with a single bout of exercise with music^3^. Therefore, in the current study, the CWST was performed before and after listening to GR while monitoring l-DLPFC activity using fNIRS. In addition to this acute model, cluster analysis using the subjective senses of both groove sensation and psychological state when listening to GR was introduced, and we tried to reveal the individual differences in the effect of listening to GR on EF and task-related l-DLPFC activity.

GR elicits groove sensation and concurrent positive affective response, but it is not known whether it enhances inhibitory EF with l-DLPFC activity as a result. The purpose of this study is to determine whether GR enhances EF and l-DLPFC activity, focusing on individual differences in psychological responses to GR. Our working hypothesis is that GR presented as drum breaks with low to medium syncopation enhances CWST performance with task-related l-DLPFC activation. Furthermore, the effects can be remarkable in participants who experience a higher groove sensation and positive psychological state. This study will allow us to look ahead to new aspects of the effect of GR, for example a potential cumulative effect with exercise.

## Results

### Physical load and psychological measures

Physical load and psychological measures for each experimental condition for all participants are shown in Table 1. Paired *t* tests were conducted over condition (WM, GR). We confirmed that there were no differences in HR between conditions (t(48) = − 0.983, *P* = 0.33). GR elicited significantly higher scores compared to WM in these items: “Good *nori*” (t(50) = − 11.99, *P* < 0.001), “Wanting to move to the music” (t(50) = − 9.17, *P* < 0.001), “Feeling like my body is resonating with the rhythm” (t(50) = − 5.62, *P* < 0.001), “Having fun” (t(50) = − 11.62, *P* < 0.001), “Excited” (t(50) = − 9.00, *P* < 0.001), and “Feeling clear-headed” (t(50) = − 4.16, *P* < 0.001). WM elicited significantly higher scores compared to GR in these items: “Struggling to synchronize with the beat” (t(50) = 4.35, *P* < 0.001), “Bored” (t(50) = 10.73, *P* < 0.001), “Wanting to stop listening” (t(50) = 6.48, *P* < 0.001), and “Feeling discomfort” (t(50) = 5.18, *P* < 0.001).

**Table 1:** Summary of results for physical load and psychological measures for all participants. Data are presented as mean (SD).

	WM condition	GR condition	*T* test
Heart rate	85.43 (13.64)	86.95 (15.75)	*P* = 0.33
**Sensation of groove**			
Good *nori*	3.35 (2.52)	7.31 (1.58)	*P* < 0.001
Wanting to move to the music	3.78 (0.37)	6.64 (0.30)	*P* < 0.001
Feeling like my body is resonating with the rhythm	3.39 (0.33)	5.09 (0.30)	*P* < 0.001
Easy to synchronize with the beat	7.07 (0.31)	6.41 (0.33)	*P* = 0.07
Struggling to synchronize with the beat	4.88 (0.42)	2.96 (0.33)	*P* < 0.001
**Psychological states**			
Having fun	3.54 (2.04)	6.66 (1.72)	*P* < 0.001
Excited	2.25 (1.90)	4.64 (2.05)	*P* < 0.001
Bored	6.11 (0.31)	2.60 (0.27)	*P* < 0.001
Wanting to stop listening	4.76 (0.36)	2.52 (0.30)	*P* < 0.001
Feeling clear-headed	5.31 (1.77)	6.45 (1.50)	*P* < 0.001
Feeling discomfort	3.00 (0.33)	1.56 (0.21)	*P* < 0.001

### Stroop task performance and l-DLPFC activity

**Figure 1:**
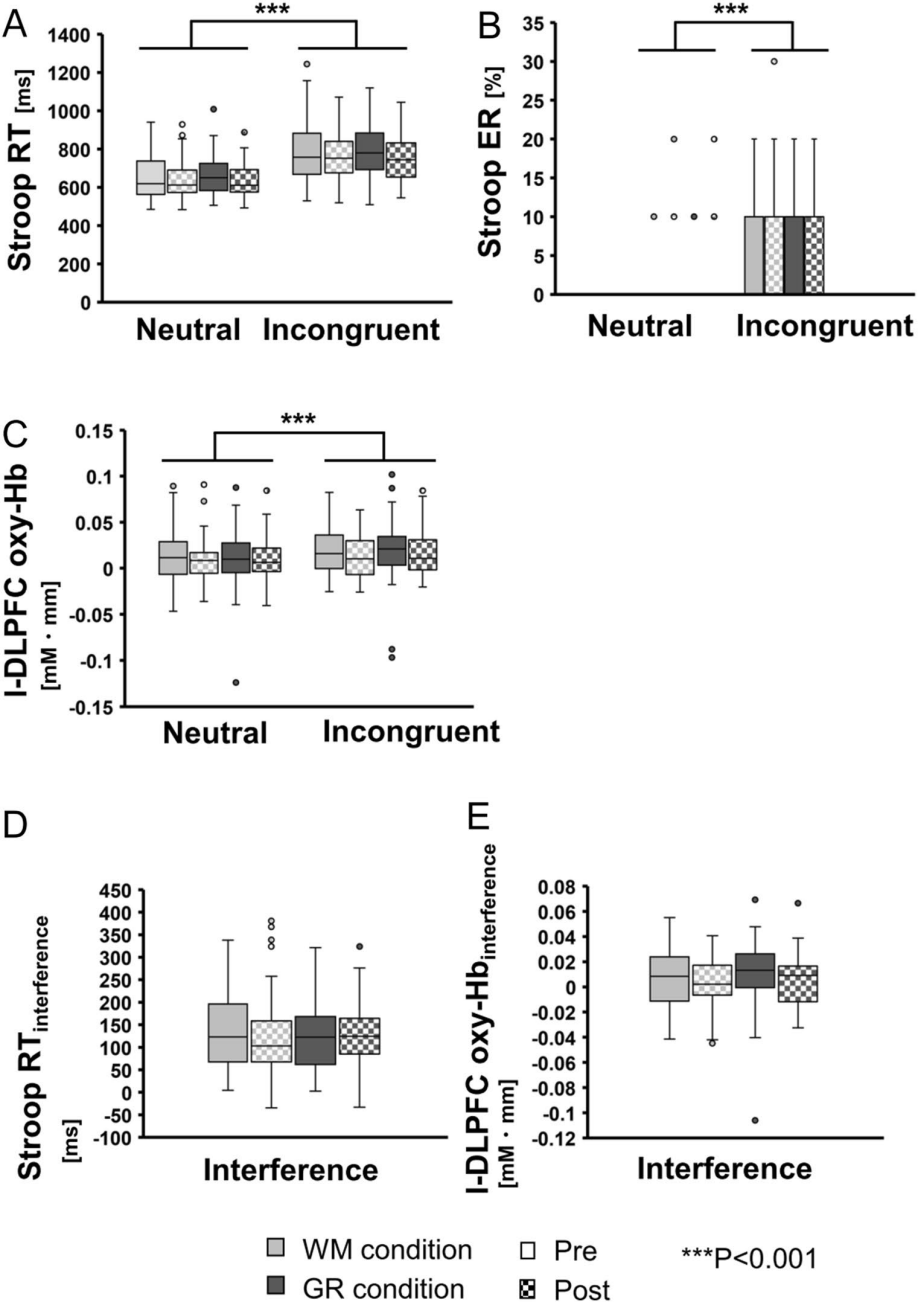
(**A**) Reaction time (RT) of neutral and incongruent conditions of the Stroop task. (**B**) Error rate (ER) of neutral and incongruent conditions of the Stroop task. (**C**) l-DLPFC oxy-Hb for neutral and incongruent conditions. Slower RT and higher ER in the incongruent condition exhibited significant Stroop interference effects. (**D**) Stroop interference against RT for the Stroop task. The repeated measures two-way ANOVA revealed no significant differences. (**E**) Stroop interference against l-DLPFC oxy-Hb. The repeated measures two-way ANOVA revealed no significant differences. Values are represented as box plots where the bottom, middle, and top lines of the boxes are the 25th, 50th (median), and 75th percentiles, respectively, and the whiskers above and below each box indicate the most extreme point within 1.5 times the interquartile range. The points above or below the whiskers represent outliers. ***P < 0.001.

We confirmed whether the Stroop interference could be observed in this experiment (Fig. 1A-C). Reaction time (RT) and error rate (ER) were subjected to a repeated measures three-way ANOVA with task condition (Neutral, Incongruent), experimental condition (GR, WM), and time (Pre, Post) being within-subject factors. The ANOVA for RT and ER exhibited significant main effects of task condition (F(1,50) = 210.06, P < 0.001, Fig. 1A, F (1,50) = 40.15, *P* < 0.001, Fig. 1B, respectively). We confirmed that the incongruent condition was more difficult than the neutral condition, and Stroop interference occurred in the present study. In the same way, for fNIRS data, l-DLPFC activity exhibited significant main effects of task condition: l-DLPFC oxy-Hb change during the incongruent condition was significantly larger than that during the neutral condition (F(1,50) = 14.80, *P* < 0.001, Fig. 1C).

Stroop RT_neutral_, RT_incongruent_, RT_interference_, l-DLPFC oxy-Hb_neutral_, l-DLPFC oxy-Hb_incongruent_, and l-DLPFC oxy-Hb_interference_ were subjected to repeated measures two-way ANOVA with condition (WM, GR) and time (Pre, Post) as within-subject factors (Fig. 1D, E). For Stroop RT_neutral_, RT_incongruent_, there was a main effect of time. Both Stroop RT_neutral_ and RT_incongruent_ were shorter for post-task than for pre-task (F(1,50) = 25.02, *P* < 0.001 and F(1,50) = 15.18, *P* < 0.001, respectively). There was no significant change of RT_interference_ (F(1,50) = 1.33, *P* = 0.25), l-DLPFC oxy-Hb_neutral_ (F(1,50) = 0.89, *P* = 0.34), l-DLPFC oxy-Hb_incongruent_ (F(1,50) = 0.78, *P* = 0.37), or l-DLPFC oxy-Hb_interference_ (F(1,50) = 0, *P* = 0.99) before or after listening for either of the GR or WM conditions.

### Subgroup analysis

To account for individual differences, we objectively classified the participants into groups using cluster analysis based on their psychological responses to listening to GR and compared the effects between inter/intra clusters. Pearson correlation coefficient between Stroop performance and all psychological variables are shown in Table 2. The representative variable which had the strongest correlation with Stroop performance in the “Sensation of groove” and “Psychological states” categories was “Good *nori*” (r = − 0.32, *P* < 0.05) and “Feeling clear-headed” (r = − 0.438, *P* < 0.01), respectively. In subsequent analyses,“Good *nori*” and “Feeling clear-headed” were used as representative items for psychological responses to listening to GR.

**Table 2:** Correlation of coefficients between Stroop performance and all psychological variables. There are two categories: “Sensation of groove” and “Psychological state”. *P < 0.05, **P < 0.01.

Category	Variable (GR − WM)	Correlation coefficient with ΔStroop RT_interference_ (GR − WM)
Sensation of groove	Good *nori*	− 0.320*
	Wanting to move to the music	− 0.142
	Feeling like my body is resonating with the rhythm	− 0.013
	Easy to synchronize with the beat	0.128
	Struggling to synchronize with the beat	− 0.094
Psychological states	Having fun	− 0.095
	Excited	− 0.288*
	Bored	0.090
	Wanting to stop listening	0.076
	Feeling clear-headed	− 0.438**
	Feeling discomfort	0.182

In the two-cluster solutions, the ratio [inter-cluster variance/total variance] was 0.41 and there was overlapping of two clusters in the plot (Supplementary Fig. S1A). The three-cluster solution was employed because of its higher ratio (0.63) and less overlapping of clusters in the plot (Supplementary Fig. S1B).

The first cluster (n = 16) was characterized by high groove and high feeling clear-headed and named the “Groove-familiar” cluster. The second cluster (n = 14) was characterized by high groove and low feeling clear-headed and named the “Low feeling clear-headed” cluster. The third cluster (n = 21) was characterized by low groove and low feeling clear-headed and named the “Groove-unfamiliar” cluster.

**Figure 2:**
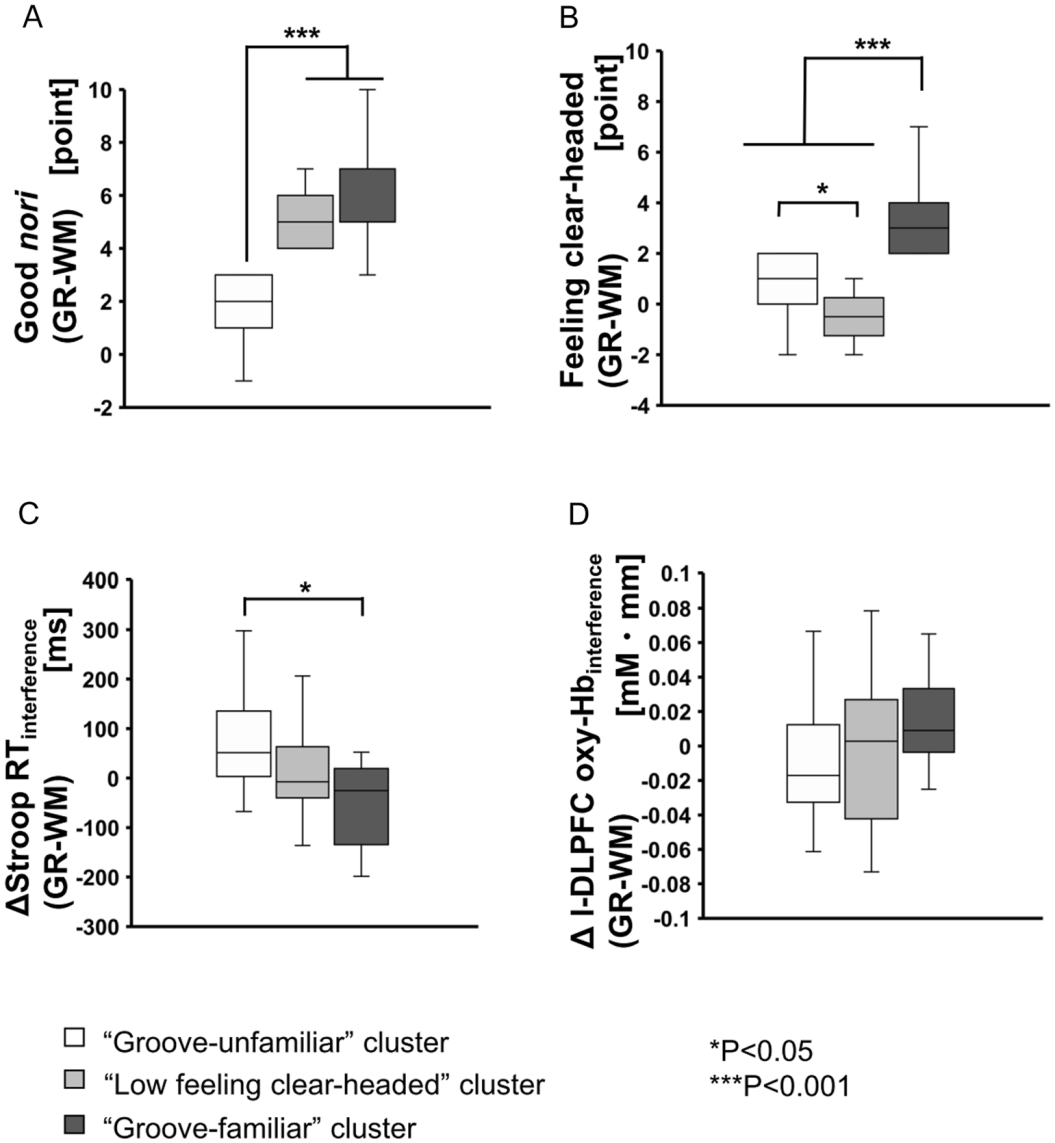
Comparison between clusters of “Good *nori*” scores, “Feeling clear-headed” scores, ΔStroop RT_interference_, and Δl-DLPFC oxy-Hb_interference_. All variables represent condition differences (GR − WM). Values are represented as box plots where the bottom, middle, and top lines of the boxes are the 25th, 50th (median), and 75th percentiles, respectively, and the whiskers above and below each box indicate the most extreme point within 1.5 times the interquartile range. The points above or below the whiskers represent outliers. ***P < 0.001, *P < 0.05.

In the comparison of condition differences (GR-WM) between clusters, a repeated measures one-way ANOVA with cluster (“Groove-familiar”, “Low feeling clear-headed”, and “Groove-unfamiliar”) was conducted (Fig. 2). The “Groove-familiar” and “Low feeling clear-headed” clusters had higher “Good *nori*” (GR-WM) than the “Groove-unfamiliar” cluster (F (2,50) = 46.15, *P* < 0.001). The “Groove-familiar” cluster had higher “Feeling clear-headed” (GR-WM) than the “Low feeling clear-headed” and the “Groove-unfamiliar” clusters (F(2,50) = 36.44, *P* < 0.001). The “Groove-unfamiliar” cluster had higher “Feeling clear-headed” (GR-WM) than the “Low feeling clear-headed” cluster (*P* < 0.05). In the comparison of basic individual specifications of music/dance between the clusters with a repeated measures one-way ANOVA, no significant differences were revealed (Supplementary Table S1).

**Figure 3:**
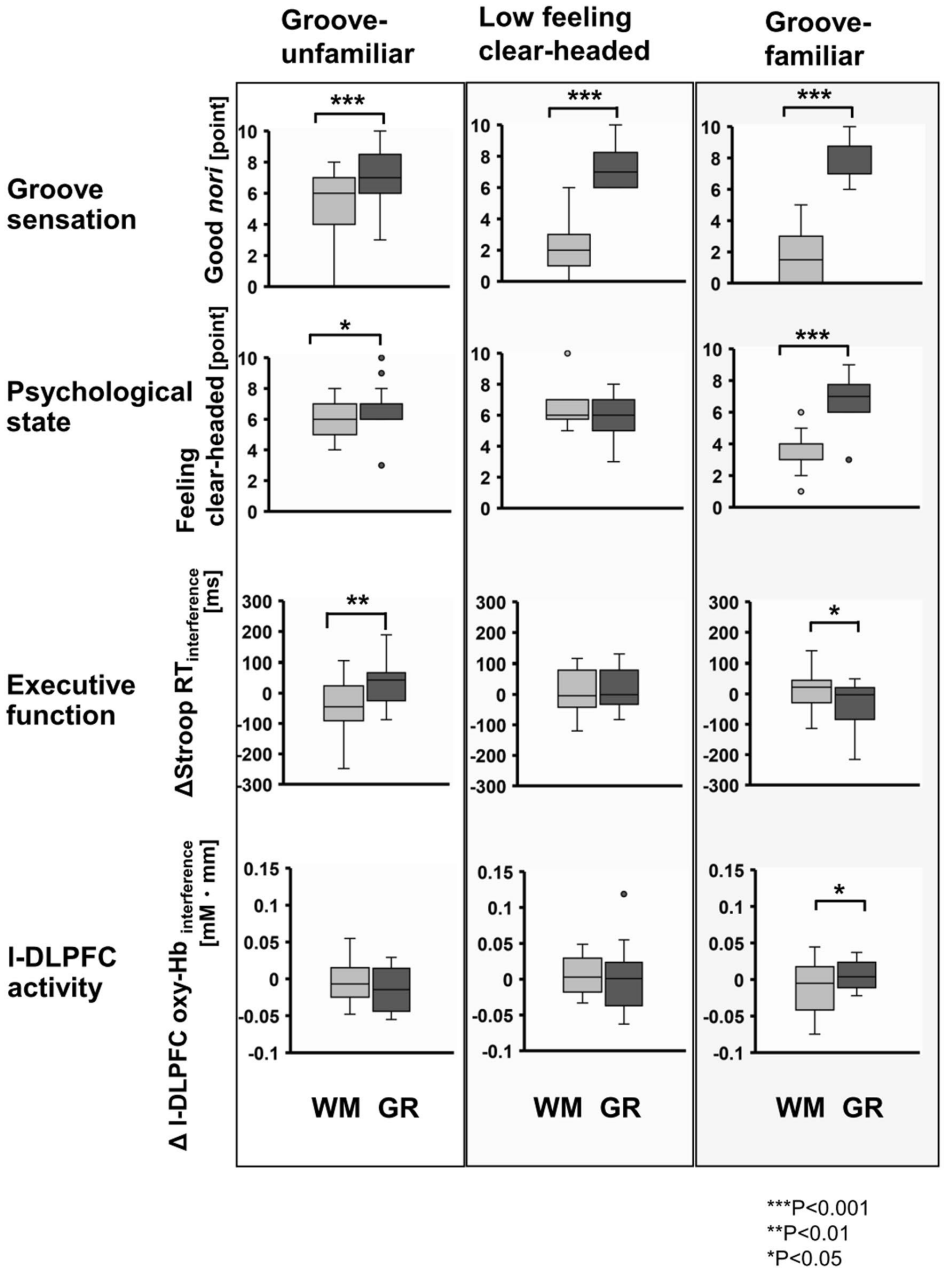
The comparison between conditions of “Good *nori*” score, “Feeling clear-headed” score, ΔStroop RT_interference_, and Δl-DLPFC oxy-Hb_interference_ within each cluster. In cluster 1 ( “Groove familiar”), ΔStroop RT_interference_ in the GR condition was shorter than in the WM condition. Conversely, in cluster 3 (“Groove unfamiliar”), ΔStroop RT_interference_ in the GR condition was longer than in the WM condition. Values are represented as box plots where the bottom, middle, and top lines of the boxes are the 25th, 50th (median), and 75th percentiles, respectively, and the whiskers above and below each box indicate the most extreme point within 1.5 times the interquartile range. The points above or below the whiskers represent outliers. ***P < 0.001, **P < 0.01, *P < 0.05.

Next, comparisons between condition (WM, GR) within each cluster were conducted using the paired *t* test (Fig. 3). In the “Groove-familiar” cluster, ΔStroop RT_interference_ for the GR condition was significantly shorter than for the WM condition (t (15) = 2.22, *P* < 0.05). Δl-DLPFC oxy-Hb_interference_ in the GR condition was significantly increased compared to the WM condition (t(15) = − 2.16, *P* < 0.05). In the “Low feeling clear-headed” cluster, there were no significant differences between conditions for both CWST performance and l-DLPFC activity. In the “Groove-unfamiliar” cluster, ΔStroop RT_interference_ in the WM condition was significantly shorter than in the GR condition (t (20) = − 3.52, *P* < 0.01). There were no significant differences between conditions for l-DLPFC activity.

### Path analysis

**Figure 4:**
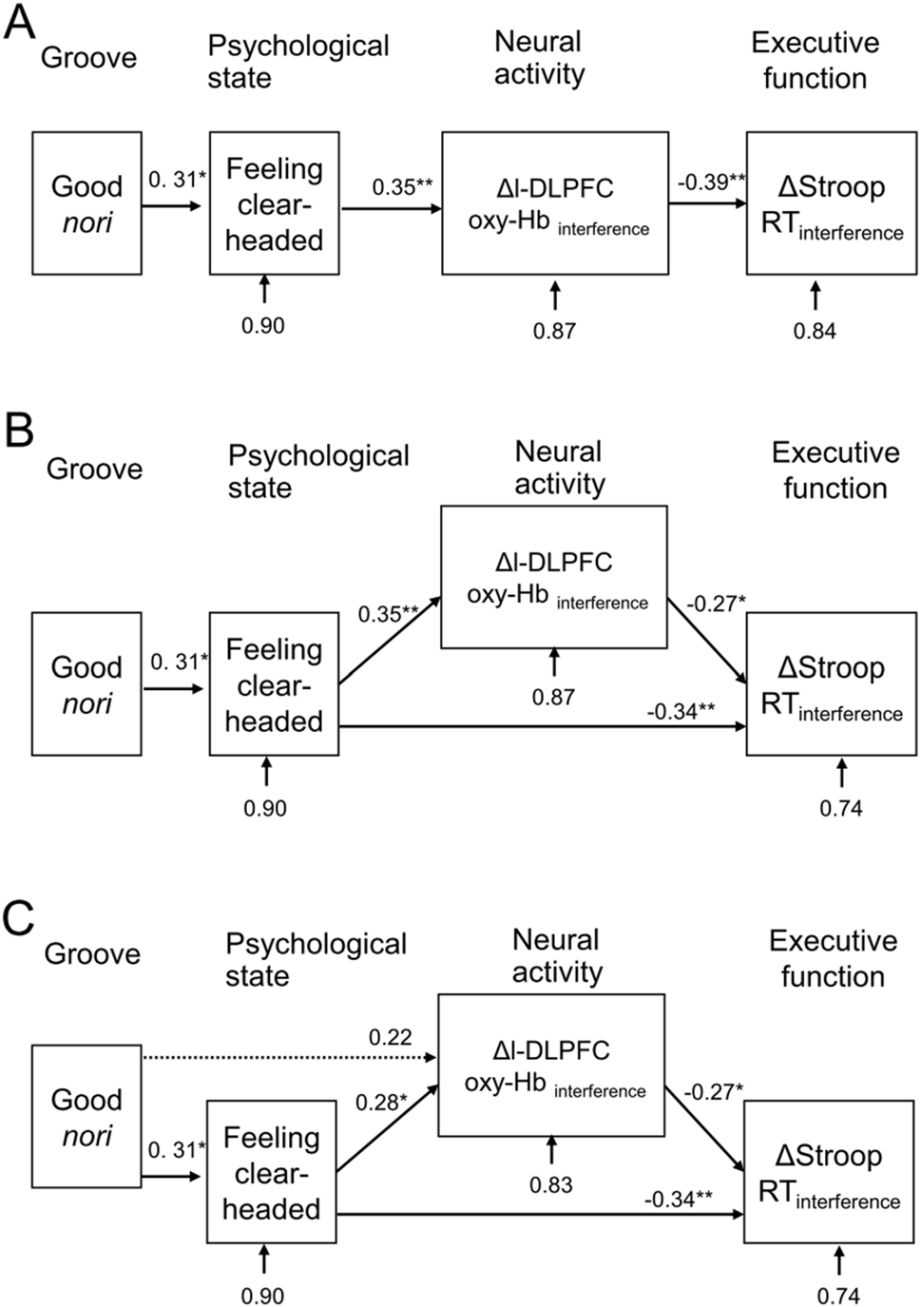
Path analyses. (**A**) The first model. (**B**) The Second model. (**C**) The third model. Variables are “Good *nori*”, “Feeling clear-headed”, Δl-DLPFC oxy-Hb_interference_, and ΔStroop RT_interference_. All variables represent condition differences (GR—WM). **P < 0.01, *P < 0.05.

In all models, the variables which were employed in cluster analysis (“Good *nori*”, “Feeling clear-headed”), Δl-DLPFC oxy-Hb_interference_, and ΔStroop RT_interference_ were used. The first model, χ^2^ = 0.01, CFI = 0.69, and RMSEA = 0.22, yielded a very poor fit to the data. The relative effects were as follows (Fig. 4A): “Good *nori*” to “Feeling clear-headed” (β = 0.31, *P* < 0.05); “Feeling clear-headed” to Δl-DLPFC oxy-Hb_interference_ (β = 0.35, *P* < 0.01); and Δl-DLPFC oxy-Hb_interference_ to ΔStroop RT_interference_ (β = − 0.39, *P* < 0.01). The second model, χ^2^ = 0.12, CFI = 0.91, and RMSEA = 0.14, yielded a poor fit to the data. The relative effects were as follows (Fig. 4B): “Good *nori*” to “Feeling clear-headed” (β = 0.31, *P* < 0.05); “Feeling clear-headed” to Δl-DLPFC oxy-Hb_interference_ (β = 0.35, *P* < 0.01); “Feeling clear-headed” to ΔStroop RT_interference_ (β = − 0.34, *P* < 0.01); and Δl-DLPFC oxy-Hb_interference_ to ΔStroop RT_interference_ (β = − 0.27, *P* < 0.05). The third model, χ^2^ = 0.24, CFI = 0.98, and RMSEA = 0.08, yielded a satisfactory fit to the data. The relative effects were as follows (Fig. 4C): “Good *nori*” to “Feeling clear-headed” (β = 0.31, *P* < 0.05); “Good *nori*” to Δl-DLPFC oxy-Hb_interference_ (β = 0.22, *P* = 0.09); “Feeling clear-headed” to Δl-DLPFC oxy-Hb_interference_ (β = 0.28, *P* < 0.05); “Feeling clear-headed” to ΔStroop RT_interference_ (β = − 0.34, *P* < 0.01); and Δl-DLPFC oxy-Hb_interference_ to ΔStroop RT_interference_ (β = − 0.27, *P* < 0.05). The final model showed both “Good *nori*” inducing “Feeling clear-headed” and Δl-DLPFC oxy-Hb_interference_ as starting factors and that “Feeling clear-headed” affected both Δl-DLPFC oxy-Hb_interference_ and ΔStroop RT_interference_.

## Discussion

The purpose of this study was to determine whether GR enhances EF and DLPFC activity, focusing on individual differences in psychological responses to GR. To achieve this purpose, the current study tested the hypothesis that GR presented as drum breaks with low to medium syncopation enhances CWST performance with task-related l-DLPFC activation, and that the effects can be remarkable in participants who show higher groove sensation and positive psychological state. To our knowledge, this study presents the first experimental evidence for the enhancing effects of GR on EF and l-DLPFC activity in only participants for whom listening to GR largely augments both groove sensation and feeling clear-headed. In addition, these psychological responses to listening to GR were shown to predict EF and l-DLPFC activity.

First, as a precondition of the experiment, we confirmed that drum breaks with a low to medium degree of syncopation (GR) induced groove sensation (e.g., “Wanting to move to the music”, “Good *nori*”) and positive affective response (e.g., “Having fun”, “Excited”) compared with the WM by comparing average values. This agreed with previous studies showing that drum breaks with a low to medium degree of syncopation induce higher groove sensation^7–9^. To make the rhythm more interesting to listeners and to thus induce groove sensation and the related positive affective responses, a balance between expectation and violation of the rhythm is thought to be important^12^. This is supported by the hypothesis that prediction error (deviation from predicted rhythm) is a requisite for music to activate the reward system in the brain^43–45^. Since the validity of our experimental design was confirmed, we proceeded to the detection of the effect of GR on cognitive function.

Regarding EF and l-DLPFC activity, we confirmed that there was Stroop interference in both Stroop task performance and l-DLPFC activity. However, there were no significant differences between experimental conditions. One possible reason for this is extensive individual differences in psychological response to GR. Thus, we conducted a sub-group analysis to consider the influences of individual differences in psychological response. Using the k-means clustering method in which “Good *nori*” and “Feeling clear-headed” were the variables, participants were divided into three clusters. The results show that only in participants who felt a high groove sensation and a high feeling clear-headed (“Groove-familiar” cluster), listening to GR significantly enhanced EF and l-DLPFC activity compared to the WM condition (Fig. 3). Conversely, GR significantly decreased EF in the participants who felt a relatively low groove sensation and a low feeling clear-headed (“Groove-unfamiliar” cluster). Furthermore, using path analysis, we detected a potential causal relationship between groove sensation, psychological state, l-DLPFC activity, and EF (Fig. 4C). The model described that groove sensation influenced both psychological state and l-DLPFC activity and that psychological state influenced EF and l-DLPFC activity. When interpreting neural activity, it should be considered together with task performance. With regard to improved task performance, neural activity may decrease, remain unchanged, or increase. A decrease or no change in neural activity despite an improvement in task performance can be interpreted as an increase in neural efficiency. Gender, task difficulty, and training are known to be influential factors of neural efficiency^46^. On the other hand, if neural activity increases with improved task performance, it can be interpreted as an increase in neural activity to achieve higher task performance. In the present study, increased l-DLPFC neural activity was positively correlated with better CWST performance (Fig. 4C), suggesting that the l-DLPFC neural activity led to higher EF. This interpretation has also been validated by other previous studies^33–35^ including our own^3,27,28^. These results suggest that groove sensation and psychological state are important predictors, and these factors influence the effect of GR not only positively but also negatively.

The participants who were grouped in the “Groove-familiar” cluster experienced positive effects of GR on EF and l-DLPFC activity. Successful entrainment and body movement to a musical beat (rhythmic entrainment, sensorimotor synchronization) could be among the important factors for promoting the positive effects of GR. Generally, music induces positive affective responses and concurrently increases dopamine release and brain activation related to the reward system including the basal ganglia (BG), midbrain, and orbitofrontal cortex^47–49^, whereas rhythmic entrainment reinforces both groove sensation and positive affective responses^18^ and recruits brain regions related to both the motor and reward systems through BG activity, which plays a key role in the connection of rhythmic entrainment and positive affective responses^19,50,51^. That groove music recruits not only the reward system, but also the motor system indicates that the body and musical entrainment are the bases of inducing a positive affective response. In addition to recruiting the dopaminergic reward system, high-groove music also induces physiological arousal related to the noradrenergic system, which is part of the catecholamine system, inducing brain activation^5^. A positive affective response involving the catecholamine system can trigger the enhancement of EF and related DLPFC activity^21–23^. Considering that both groove sensation and positive affective responses were together correlated with BG activity in a previous study^11^ and that the current data shows that both groove sensation and feeling clear-headed are influential factors in the effect on l-DLPFC activity and EF, we could postulate a relationship between rhythmic entrainment, psychological responses, the catecholamine system, and prefrontal function. Thus, “Groove-familiar” participants may have achieved successfully entrainment to GR, enhanced groove sensation and positive psychological states, and these psychological response may have triggered the release of neurotransmitters resulting in l-DLPFC activation and EF enhancement.

Conversely, the participants grouped in the “Low feeling clear-headed” and “Groove-unfamiliar” clusters experienced no or negative effects of GR on EF and l-DLPFC activity. One potential inhibiting factor is low beat-processing ability in those individuals. It is possible that participants who have a low beat-processing ability were forced to pay extra attention to the beat while listening to the GR. This may have resulted not only in inducing failure to become entrained, but also in reduced mental resources, which limited attention, motivation, and cognitive performance^52–54^. In the current experiment, the average scores for the beat-processing ability test among participants in both the “Low feeling clear-headed” and "Groove-unfamiliar" clusters were relatively low compared to the participants in the "Groove-familiar" cluster, but not significantly so (Supplementary Table S1). Since the beat alignment test used in the current study was a simple battery test to evaluate beat processing ability, further studies using battery tests with a higher sensitivity (e.g., H-BAT^55^) are needed to detect the influence of inhibiting factors.

This study has several limitations. First, widespread individual differences in psychological responses to the rhythm were demonstrated in the current results. Various potential factors such as musical training experience, music reward sensitivity, beat processing ability, familiarity with groove music/dance, body morphology and cultural background could influence psychological responses to the rhythm^7,8,15,56–58^. Though more than 50 students participated in the current experiment, the sample size was insufficient for conducting sub-analyses to seek the effects of these potential influential factors. Future studies should plan to compare the effects between participants grouped by these potential influential factors. Second, in the auditory stimulus, we used rhythm with a low to medium degree of syncopation as the GR stimulus and a white-noise metronome as the control stimulus. We could control tempo, but could not control the type and number of sounds. Further studies are needed to try to detect the influence of other acoustic specifications by using different patterns for the GR and control stimuli. Third, in the measurement of the brain’s neural activation, the current study focused on only cortical, specifically the l-DLPFC, activity. We should use fMRI and PET to examine brain activity in broader and deeper regions that are involved with motor, reward, and cognitive systems, their networks, and released neurotransmitters.

In conclusion, listening to a rhythm with low to medium syncopation enhanced EF and l-DLPFC activity in only groove-familiar participants, which supports our hypothesis. These results suggest that individual differences in psychological responses to GR are one of the key factors in predicting the effects of listening to GR on prefrontal EF. This study raises the potential that GR can enhance human cognitive performance like exercise.

## Materials and methods

### Participants

Fifty-eight healthy young adults (mean age 20.19 ± 1.84 years, 28 female) took part in this study. Our previous fNIRS studies examining the effect of very light to moderate intensity exercise on CWST performance^27,28^ showed that the effect sizes of exercise were Cohen's d = 0.5 ~ 0.7. In the current study, we estimated a slightly lower effect of GR than of exercise and conducted a Power Analysis with Cohen's d = 0.4. A Power Analysis using the sample size determination software G-power showed that 52 subjects would be considered sufficient to detect a significant difference (dz = 0.9) between groups on a two-sided, 0.05 test of proportions (difference between two dependent means [matched pairs]) with > 80% power. All participants were recruited from the student population of the University of Tsukuba through ethics board approved flyers that were displayed across the campus. No subject reported a history of neurological or psychiatric disorders or had a disease requiring medical care. All participants were Japanese native speakers and right-handed. All experimental protocols were approved by the Institutional Ethics Committee of the University of Tsukuba. The experiment protocols were in accordance with the guidelines of the latest version of the Helsinki Declaration. Written informed consent was obtained from all participants after being given complete information about this study. Before all statistical processes, seven participants were excluded from the analysis for the following reasons: reported short sleep duration the night before the experiment (N = 1), had a pre-session CWST correct-answer rate equal to 80% or less (N = 1), had a pre-session Stroop RT_interference_ with a negative value or 2SD greater than the average (N = 3), or had missing fNIRS data (N = 2). As a result, we analyzed the data of fifty-one participants. Demographics of the participants are presented in Supplementary Table S2. All participants were requested to abstain from intense exercise and the consumption of alcohol and caffeine for at least 24 h prior to each experiment so as to control for outside factors that could affect EF.

### Experimental procedure

**Figure 5:**
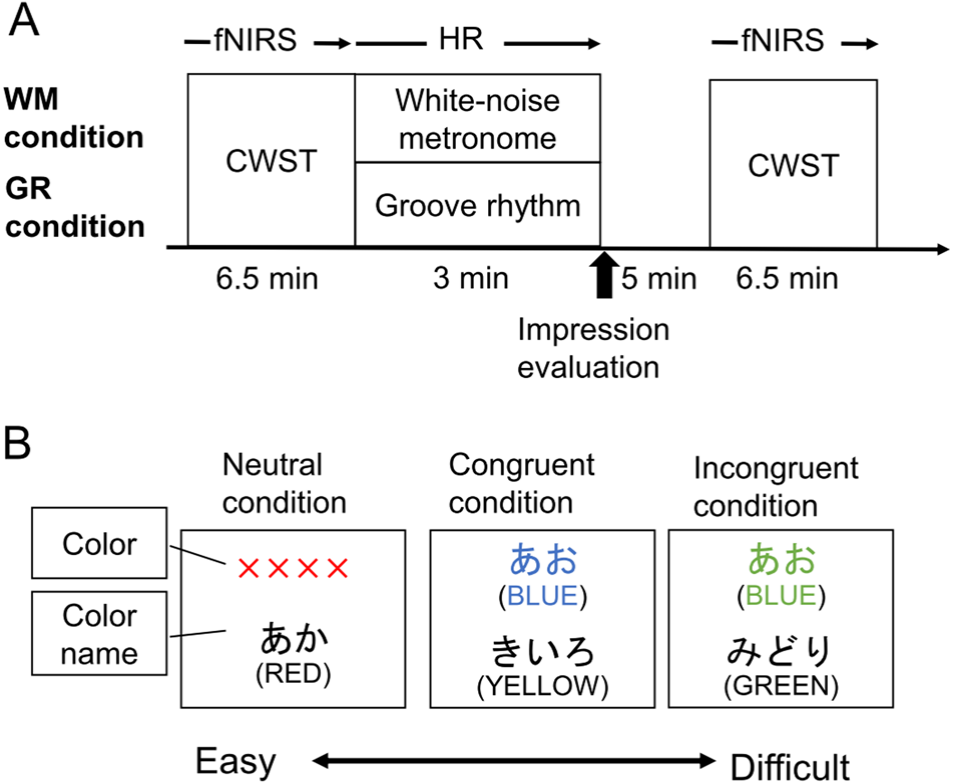
(**A**) Experimental protocol. (**B**) Color-word Stroop task (CWST). There are three conditions: neutral, congruent, and incongruent. In incongruent trials, interference occurs and creates a delayed reaction time compared to neutral trials because in the top row, the word meaning does not match the ink color. We calculated the differences in reaction time for incongruent minus neutral conditions to determine Stroop interference time (Stroop RT_interference_), which represents executive function.

The overall procedures consisted of four visits for participants. On the first day, the participants filled out questionnaires about demographics and practiced the CWST. On the second day, to measure basic individual features of music/dance, they performed a beat alignment test, which consists of judgement and tapping tests^59^, and they rated their dance familiarity by responding to the phrase, “I often start to dance when music plays” on an eleven-point scale ranging from 0 = “Not at all”, 5 = “Neutral” and 10 = “Extremely”. On the third and fourth days, they underwent two experimental conditions: listening to groove rhythm (GR condition) or listening to a white-noise metronome (WM condition). The participants listened to auditory stimuli played through studio speakers (HR824mk2, Mackie) for 3 min in a standing position. To increase the groove sensation^18^, participants were instructed to “please beat the rhythm freely in your upper body, for example, tapping, nodding, and swaying”. However, dynamic physical movement such as stepping was discouraged. In order to confirm the physiological load during listening, heart rate (HR) was measured using a wireless sports heart rate meter (Polar V800, Polar). Before and after listening, the CWST was conducted while hemodynamic changes in the l-DLPFC were monitored using fNIRS. After listening, participants answered a questionnaire about their psychological responses to the stimuli (e.g., “Good *nori*”; “Feeling clear headed”) (Fig. 5A). The order of conditions was randomized. Both conditions were conducted at the same time of day for each participant. All visits were separated by at least 48 h. The details of the measurements are described below.

### Auditory stimuli

A drum break with a low to medium degree of syncopation was used and was about 3 min long. The drum break was created using the software “GarageBand” (Apple, Inc.) by referencing a previous study^60^ (Supplementary Table S3). In order to control the degree of syncopation and low-frequency components, we prepared a white-noise metronome as the control rhythm with a zero degree of syncopation and a flat spectrum over the audible frequency range using the free software “Audacity” (Audacity team). The tempo of the rhythm was set at 120 bpm because around 100–120 bpm is an appropriate tempo for inducing groove with drum beats^6^.

### Psychological measures

To evaluate the potential for a psychological response to listening to the rhythm to mediate the effects on EF, we created two categories each consisting of eleven questions. The first category was about the sensation of groove including “Good *nori*”, “Wanting to move to the music”, “Feeling like my body is resonating with the rhythm”, “Easy to synchronize with the beat”, and “Struggling to synchronize with the beat”. We employed several terms closely related to groove as suggested in previous studies^6,61^. “Good *nori*” is a Japanese term similar to the concept of groove^6^. These items were also shown to be highly correlated with groove. The second category was about psychological state and included descriptors such as “Having fun”, “Excited”, “Bored”, “Wanting to stop listening”, “Feeling clear-headed”, and “Feeling discomfort”. “Having fun” and “Excited” have been shown to correlate with groove in previous studies^6,62^. “Bored”, “Wanting to stop listening”, and “Feeling discomfort” were the reverse. “Feeling clear-headed” was set as an item which expresses cognitive alertness^63,64^ to detect responses to GR based on the preliminary study. The eleven-point (0–10) scale ranged from 0 = “Not at all”, 5 = “Neutral” to 10 = “Extremely”.

### Behavioral measures

The Japanese version of the CWST, which has been validated in previous studies, was used to assess inhibitory control of a core component of executive function (Fig. 5B)^3,27,28^. The CWST is a task in which participants are presented with two rows of letters and they must determine whether the top row’s color corresponds to the bottom row’s color name. Subjects answered by pressing the "yes" or "no" buttons on a keyboard ("C" and "N", respectively) with the index finger of their right and left hands.

This task consists of three conditions: neutral, congruent, and incongruent. In the lower part of the screen, one of the four color-name words "RED", "BLUE", "GREEN", and "YELLOW" were displayed, in Japanese, for all trials. However, the characters displayed in the upper row of the screen were different for each condition: the symbol "xxxx" was presented in the neutral condition, a color word written in an ink color matching its meaning (e.g., the word “BLUE” written in blue ink) was presented in the congruent condition, and a color word displayed in a color that did not match its meaning (e.g., the word “BLUE” written in yellow ink) was presented in the incongruent condition. The incongruent condition causes a cognitive conflict between the meaning and color of the upper row’s word and induces a delay in reaction time (RT). We randomly displayed 30 trials including 10 neutral, 10 congruent, and 10 incongruent.

To force the subjects to look from the upper row to the lower row, the upper row was displayed 100 ms earlier than the lower row. After 2 s of the upper characters being displayed, the screen returned to the fixation screen, which was centered on a "+" mark (gazing point). The inter-stimulus interval was randomized (10–12 s) for each subject to prevent them from predicting the timing. We evaluated RT for the incongruent task minus that for the neutral task to determine Stroop interference time (Stroop RT_interference_), which represents inhibitory executive function. Every participant practiced the CWST at least three times before starting the first CWST session.

### fNIRS data acquisition

**Figure 6:**
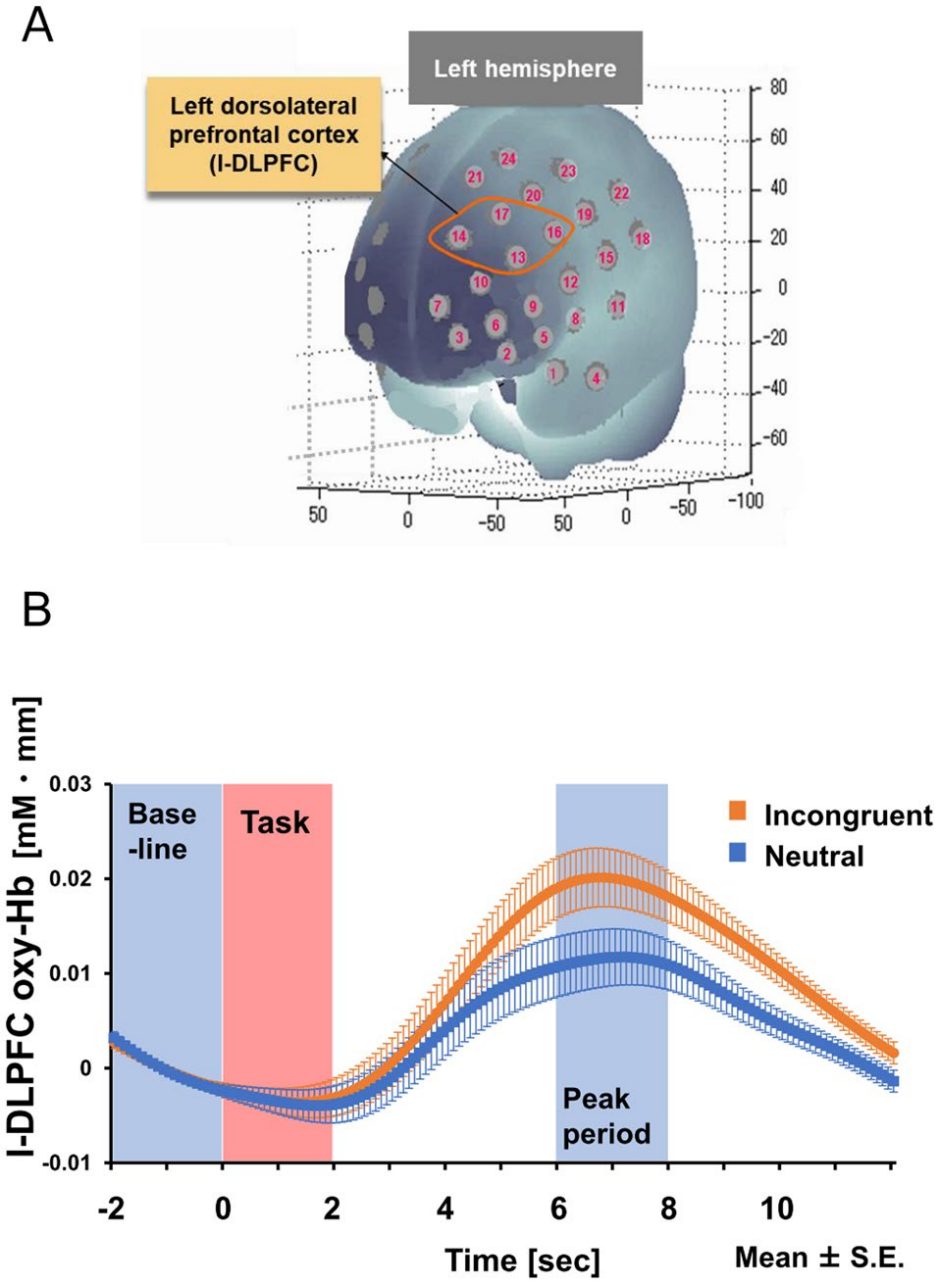
(**A**) The spatial profiles of fNIRS channels and ROI segmentation. (**B**) Time course of l-DLPFC oxy-Hb change (average of all Pre sessions). From − 2 to 0 is the baseline period, from 0 to 2 is the Task period, and from 6 to 8 is the peak period. The differences between incongruent trials (orange line) and neutral trials (blue line) at the peak period were evaluated as Stroop oxy-Hb_interference_.

To measure hemodynamic changes in the l-DLPFC, the multichannel fNIRS optical system ETG-700 (Hitachi Medical Corporation, Japan), which is equipped with two wavelengths of near-infrared light (785 and 830 nm), was used. To cover the l-DLPFC using an fNIRS probe holder, the composition and placement of the fNIRS probe holder followed the same procedure as described in our previous studies^3,27–31^. A set of two 4 × 4 multichannel probe holders was placed over the prefrontal region according to virtual registration. The left probe holder was placed such that probe 5 (between CH 4 and CH 11) was placed over FT7, with the medial edge of the probe column parallel to the medial line (Fig. 6A). Likewise, the right probe holder was symmetrically placed on the right hemisphere. The sampling rate was set at 10 Hz.

### fNIRS data analysis

The oxygenated hemoglobin (oxy-Hb) was analyzed and calculated in units of millimolar-millimeter (mM⋅mm)^65^. The oxy-Hb changes in the l-DLPFC were preprocessed with a band pass filter (high-pass: 0.04 Hz, low-pass: 0.3 Hz). The differences of the oxy-Hb signal between peak (6–8 s after trial onset) periods and baseline (0–2 s before trial onset) periods were calculated as the oxy-Hb change (Fig. 6B). As in previous studies^27–31^, a virtual registration method was employed to register fNIRS data to Montreal Neurological Institute (MNI) space^66,67^. In brief, this method allows us to place a virtual probe holder on the scalp using a simulation of the holder’s deformation and to register the probes and channels on reference brains in the MRI database^68–71^. ROIs are created by combining four neighboring channels, and anatomically labeled using LBPA40, which is a widely used method (Shattuck et al., 2008). The l-DLPFC as a ROI accounts for channels 13, 14, 16, and 17.

### Outline of statistical analysis

We performed three analyses to verify our hypothesis. First, we compared the average of all participants between conditions (see below, “Comparison of overall participants”). Second, we objectively classified the participants into groups using cluster analysis based on their psychological responses to listening to GR and compared the effects (see below, “Cluster analysis”). Third, we performed path analysis to estimate the potential causal relationship between parameters including Stroop task performance, cortical activation in the l-DLPFC, and psychological variables which had the highest correlation with ΔStroop RT_interference_ in the categories of “Sensation of groove” and “Psychological state” (see below, “Path analysis”). SPSS version 22 (SPSS, Inc., USA) and R software was used for all statistical processes. Statistical significance was set at *P* < 0.05.

### Comparison of overall participants

For HR and each psychological measure, paired *t* tests were conducted for condition (WM, GR). Regarding HR data, two datasets were missing because of slippage of the sensor band. For Stroop task performance, RT and ER were subjected to repeated-measures three-way ANOVA with condition (WM, GR), time (pre, post), and task condition (neutral, incongruent) as within-subject factors to examine whether the general tendencies for the Stroop task could be reproduced in all conditions. This analysis was limited to the main effect of the task condition because the purpose of the ANOVA was to examine the occurrence of Stroop interference. Subsequently, Stroop RT_neutral_, RT_incongruent_, and RT_interference_ [Incongruent—Neutral] were calculated, and repeated-measures two-way ANOVA were performed with condition (WM, GR) and time (pre, post) as within-subject factors. When there were significant interaction or main effects in the two-way ANOVA, post-hoc tests using the Bonferroni method were carried out. For l-DLPFC activity, in order to confirm whether there was a Stroop effect (l-DLPFC oxy-Hb_interference_), l-DLPFC oxy-Hb changes were subjected to repeated-measures three-way ANOVA with condition (WM, GR), time (pre, post), and task condition (neutral, incongruent) as within-subject factors as for the Stroop task performance analysis. Subsequently, l-DLPFC oxy-Hb_interference_ [Incongruent − Neutral] was calculated, and a two-way ANOVA was performed with condition (WM, GR) and time (pre, post) as within-subject factors. When there were significant interactions or main effects in the two-way ANOVA, post-hoc tests using the Bonferroni method were carried out.

Subsequently, $\Delta$Stroop RT_interference_ and $\Delta$l-DLPFC oxy-Hb_interference_ were calculated for the following contrast: [post − pre]. Condition differences of psychological measures, $\Delta$Stroop RT_interference_, and $\Delta$l-DLPFC oxy-Hb_interference_ were calculated for the following contrast: [GR-WM]. Pearson correlation analysis between all psychological measures as independent variables and $\Delta$ Stroop RT_interference_ as a dependent variable was carried out.

### Cluster analysis

The K-means method was used to identify relatively homogenous participant groups on R software. As with a previous study using the k-means method^72^, representative variables which had the highest correlation with ΔStroop RT_interference_ in each category of “Sensation of groove” and “Psychological state” were used for cluster analysis. As a result, “Good *nori*” and “Feeling clear-headed” were used. Regarding determination of the optimal number of clusters, we used the ratio of inter-cluster variance and total variance (R script: km$betweenss/km$totss)^73^ and visual inspection of the scatterplots. The ratio shows aggregability of intra-cluster and separation of inter-cluster. We determined the cluster number which showed a greater ratio of inter-cluster variance and total variance not exceeding three cluster numbers to avoid the difficulty of defining the clusters.

After clustering, to reveal the specification of clusters, the comparison of condition differences [GR-WM] in psychological measures, $\Delta$Stroop RT_interference_ and $\Delta$l-DLPFC oxy-Hb_interference_ between clusters, and the comparison of basic individual features of music/dance between clusters were conducted using a repeated measures one-way ANOVA with clusters. When there were significant main effects in the one-way ANOVA, a post-hoc test using the Gabriel method was carried out. Then, to detect the effect of listening to GR by cluster, the comparison of psychological measures, $\Delta$Stroop RT_interference_, and $\Delta$l-DLPFC oxy-Hb_interference_ between conditions within each cluster were conducted using the paired *t* test over condition (WM, GR).

### Path analysis

To address a possible causal relationship, path analysis was conducted using the “lavaan” package in R software. Model fit was evaluated with the χ^2^ goodness of fit, the root mean square error of approximation (RMSEA), and the Comparative Fit Index (CFI). The criteria were as follows: CFI ≥ 0.90, RMSEA < 0.10, and χ^2^ ≥ 0.05. Regarding RMSEA values, 0.05 is often used as the cutoff; however, this rejects too many valid models with small sample sizes (n ≤ 100)^74^. Conversely, RMSEA values greater than or equal to 0.10 suggest a poor fit^75^. Thus, we set the criteria as RMSEA < 0.10. The variables were “Feeling clear-headed”, "Good *nori*", $\Delta$l-DLPFC oxy-Hb_interference_, and $\Delta$Stroop RT_interference_, and they were reflected in differences between conditions [GR – WM].

Three models were tested to examine the causal relationship between the variables depending on the hypothesis. The first model assumed that all variables have only a direct effect. The second model allowed that “Feeling clear-headed” has two paths: to Δl-DLPFC oxy-Hb_interference_ as an indirect effect on EF and to $\Delta$Stroop RT_interference_ as a direct effect on EF. The third model was a modified version of the second model which included the influence of “Good *nori*” on Δl-DLPFC oxy-Hb_interference_.

## Supplementary Information

Supplementary Information.

## Data Availability

The datasets generated during and/or analyzed during the current study are available from the corresponding author on reasonable request.
