# Supplementary material for: Groove rhythm stimulates prefrontal cortex function in groove enjoyers
Source: Sci Rep. 2022 May 5;12:7377. doi: 10.1038/s41598-022-11324-3 (PMC9072545; doi:10.1038/s41598-022-11324-3)
Supplement: Supplementary file 1 — Supplementary Information. [file 41598_2022_11324_MOESM1_ESM.docx]

**Supplementary data**

Groove rhythm stimulates prefrontal cortex function in groove enjoyers

Takemune Fukuie^1^, Kazuya Suwabe^1,2,3^, Satoshi Kawase^4^, Takeshi Shimizu^5^, Genta Ochi^1,2,6^, Ryuta Kuwamizu^1^, Yosuke Sakairi^2^, & Hideaki Soya^1,2^*****

^1^Laboratory of Exercise Biochemistry and Neuroendocrinology, Faculty of Health and Sport Sciences, University of Tsukuba, Ibaraki 305-8574, Japan

^2^Sports Neuroscience Division, Department of Mind, Advanced Research Initiative for Human High Performance (ARIHHP), Faculty of Health and Sport Sciences, University of Tsukuba, Ibaraki 305-8574, Japan

^3^Faculty of Health and Sport Sciences, Ryutsu Keizai University, Ibaraki 301-8555, Japan

^4^Faculty of Psychology, Kobe Gakuin University, Hyogo 651-2180, Japan

^5^School of Information and Communication, Meiji University, Tokyo 101-8301, Japan

^6^Department of Health and Sports, Niigata University of Health and Welfare, Niigata 950-3198, Japan

**Table S1.** Comparison of basic individual specifications of music/dance between clusters

|  | **“Groove-familiar”**  **cluster** | **“Low feeling clear-headed”**  **cluster** | **“Groove-unfamiliar”**  **cluster** | **F-value** | **P-value** | |
| --- | --- | --- | --- | --- | --- | --- |
| **Individual specification of music/dance** | | | | | |  |
| Beat processing ability (Judgement) | 89.75 (11.19) | 83.13 (12.86) | 87.91 (10.61) | F(2, 50) =1.34 | p=0.27 | |
| Beat processing ability (Tapping) | 96.79 (4.59) | 93.88 (5.96) | 93.54 (5.96) | F(2, 50) =1.54 | p=0.22 | |
| Dance familiarity | 3.68 (2.86) | 3.14 (2.95) | 2.45 (2.66) | F(2, 50) =0.54 | p=0.58 | |

**Table S2.** Demographics of participants. Values are shown as mean (SD). Note: BMI = Body Mass Index; BDI = Beck Depression Inventory.

| **Measure** | **All** |
| --- | --- |
| Sample Size | 51 (28 female) |
| Age [yr] | 20.19 (1.84) |
| Height [cm] | 163.61 (8.00) |
| Weight [kg] | 54.37 (8.60) |
| BMI [kg/m^2^] | 20.19 (1.91) |
| BDI-2 | 6.78 (5.07) |
| Beat processing ability (Judgement) | 87.25 (11.52) |
| Beat processing ability (Tapping) | 94.76 (5.63) |
| Dance familiarity | 3.13 (2.78) |

**Table S3.** Details of GR. We made an approximately three-minute drum break by combining drum patterns which were showed in a previous study (Witek et al., 2015).

| The number of drum breaks sample  (Witek et al., 2015) | Duration | Time course | Syncopation degree | Number of snares | Number of basses |
| --- | --- | --- | --- | --- | --- |
| 4 | 32" | 0”-16” | 7 | 16 | 24 |
|  |  | 16”-32” | 7 | 16 | 24 |
| 9 | 32" | 32”-48” | 13 | 20 | 40 |
|  |  | 48”-64” | 13 | 20 | 40 |
| 3 | 32" | 64”-80” | 6 | 12 | 20 |
|  |  | 80”-96” | 6 | 12 | 20 |
| 24 | 32" | 96”-112” | 29 | 12 | 24 |
|  |  | 112”-128” | 29 | 12 | 24 |
| 26 | 48" | 128”-144” | 34 | 8 | 9 |
|  |  | 144”-160” | 34 | 8 | 9 |
|  |  | 160”-176” | 34 | 8 | 9 |

A


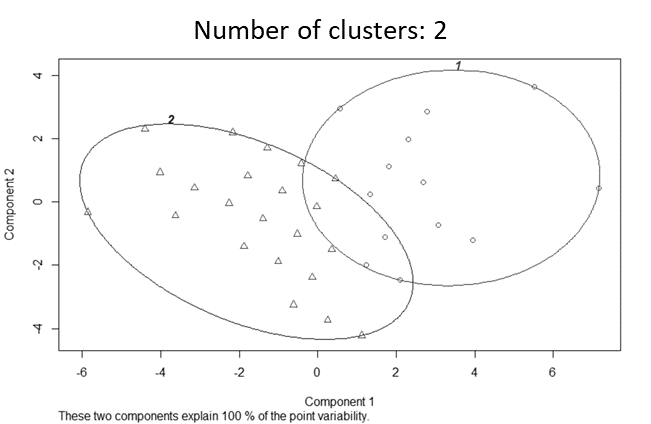


B


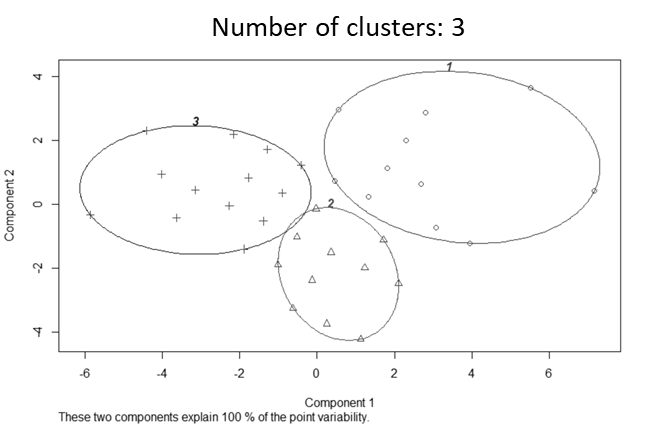


**Fig. S1. (A)** Cluster plots of the two-cluster solution. Circle number one shows the high “Good *nori*” and high “Feeling clear-headed” groups. Circle number two shows the low “Good *nori*” and low “Feeling clear-headed” groups. There was overlapping of two clusters in the plot. **(B)** Cluster plot of the three-cluster solution. Circle number one shows the high “Good *nori*” and high “Feeling clear-headed” groups (“Groove-familiar” cluster). Circle number two shows the middle “Good *nori*” and low “Feeling clear-headed” groups (“Low feeling clear-headed” cluster). Circle number three shows the low “Good *nori*” and low “Feeling clear-headed” groups (“Groove-unfamiliar” cluster). The horizontal and vertical axes of the cluster plot shown in Fig. S1 represent the first and second principal components, respectively, and do not correspond exactly to the “groove sensation” and “psychological state” used as variables.
